# Evaluating the Quality and Understandability of Radiology Report Summaries Generated by ChatGPT: Survey Study

**DOI:** 10.2196/76097

**Published:** 2025-08-27

**Authors:** Alexis Sunshine, Grace H Honce, Andrew L Callen, David A Zander, Jody L Tanabe, Samantha L Pisani Petrucci, Chen-Tan Lin, Justin M Honce

**Affiliations:** 1 Department of Radiology, University of Colorado Anschutz Medical Campus, 19th Ave. Mail Stop C278, Aurora, CO, 80045, United States, 1 303-724-3796, 1 303-724-3795; 2 Hartway Evaluation Group, Denver, CO, United States; 3 Department of Medicine, University of Colorado Anschutz Medical Campus, Aurora, CO, United States

**Keywords:** large language models, artificial intelligence, ChatGPT, radiology reports, patient-centered care, neuroradiology, report summarization

## Abstract

**Background:**

Radiology reports convey critical medical information to health care providers and patients. Unfortunately, they are often difficult for patients to comprehend, causing confusion and anxiety, thereby limiting patient engagement in health care decision-making. Large language models (LLMs) like ChatGPT (OpenAI) can create simplified, patient-friendly report summaries to increase accessibility, albeit with errors.

**Objective:**

We evaluated the accuracy and clarity of ChatGPT-generated summaries compared to original radiologist-assessed radiology reports, assessed patients’ understanding and satisfaction with the summaries compared to the original reports, and compared the readability of the original reports and summaries using validated readability metrics.

**Methods:**

We anonymized 30 radiology reports created by neuroradiologists at our institution (6 brain magnetic resonance imaging, 6 brain computed tomography, 6 head and neck computed tomography angiography, 6 neck computed tomography, and 6 spine computed tomography). These anonymized reports were processed by ChatGPT to produce patient-centric summaries. Four board-certified neuroradiologists evaluated the ChatGPT-generated summaries on quality and accuracy compared to the original reports, and 4 patient volunteers separately evaluated the reports and summaries on perceived understandability and satisfaction. Readability was assessed using word count and validated readability scales.

**Results:**

After reading the summary, patient confidence in understanding (98%, 116/118 vs 26%, 31/118) and satisfaction regarding the level of jargon/terminology (91%, 107/118 vs 8%, 9/118) and time taken to understand the content (97%, 115/118 vs 23%, 27/118) substantially improved. Ninety-two percent (108/118) of responses indicated the summary clarified patients’ questions about the report, and 98% (116/118) of responses indicated patients would use the summary if available, with 67% (79/118) of responses indicating they would want access to both the report and summary, while 26% (31/118) of responses indicated only wanting the summary. Eighty-three percent (100/120) of radiologist responses indicated the summary represented the original report “extremely well” or “very well,” with only 5% (6/120) of responses indicating it did so “slightly well” or “not well at all.” Five percent (6/120) of responses indicated there was missing relevant medical information in the summary, 12% (14/120) reported instances of overemphasis of nonsignificant findings, and 18% (22/120) reported instances of underemphasis of significant findings. No fabricated findings were identified. Overall, 83% (99/120) of responses indicated that the summary would definitely/probably not lead patients to incorrect conclusions about the original report, with 10% (12/120) of responses indicating the summaries may do so.

**Conclusions:**

ChatGPT-generated summaries could significantly improve perceived comprehension and satisfaction while accurately reflecting most key information from original radiology reports. Instances of minor omissions and under-/overemphasis were noted in some summaries, underscoring the need for ongoing validation and oversight. Overall, these artificial intelligence–generated, patient-centric summaries hold promise for enhancing patient-centered communication in radiology.

## Introduction

The 21st Century Cures Act increased patients’ access to their medical information [1,2]. This change reflected a growing desire among patients to engage with their care, a desire that is exaggerated in the field of radiology, where patients want access to reports and images, and access these materials at higher rates than general patient portals or clinical notes [3-6]. Unfortunately, radiology reports, which are primarily written for referring clinicians, use complex medical terminology that is well above the established guidelines for patient-friendly material [1-4,7-9]. Limited comprehension of these reports can leave patients feeling disempowered, anxious, and uncertain about their diagnoses and treatment plans [4]. This is particularly worrisome given that patient engagement with and comprehension of their health care information correlates with improved patient experience, improved outcomes, and decreased costs [1,3,8,10-12]. This evolving medical landscape necessitates urgent adaptations within radiology to provide easily understandable, patient-friendly reports [1,2].

There have been several attempts at improving the readability of radiology reports. For example, the University of Pennsylvania’s PORTER platform is a structured reporting system that provides automated lay-language translation of reports, and University of California, San Diego, has implemented a consumer health vocabulary automated translation and embedded hyperlinks for defining key terms [4,13,14]. However, there is a need to find ways to simplify language and avoid jargon to facilitate clear patient communication [4,8,9,9,15,16]. Radiologists face increasing workloads, making the manual preparation of simplified reports for every patient impractical [17]. Recent improvements in artificial intelligence (AI), particularly large language models (LLMs) such as ChatGPT (OpenAI), have emerged as a promising solution for generating accessible and context-appropriate textual summaries [2]. These models show promise in efficiently processing complex medical information and producing coherent summaries that minimize technical jargon while preserving essential clinical content [18,19].

The ability of LLMs to generate patient-centric summaries may help address critical barriers to patient engagement, specifically low health literacy levels and limited health care professional time for conversational patient education [8,12]. Automating this process with an LLM offers a potential solution that could be integrated into clinical workflows while preserving radiologists’ time for critical interpretive tasks. However, important concerns persist regarding the completeness and accuracy of LLM-generated outputs, biases in the underlying training data, and the consequences of generating misleading clinical information. Therefore, effective deployment of such technology requires rigorous assessment of, and balance between, the quality and simplicity of these summaries, as well as their actual benefit to patients [20,21].

This study evaluates ChatGPT’s capacity to produce patient-centered radiology report summaries through three primary aims: (1) assessing the technical accuracy and completeness of the summaries by a radiologist, (2) measuring improvements in patient comprehension and satisfaction, and (3) quantifying changes in readability using validated metrics. Through this evaluation, we aimed to provide insights into how LLM-driven text generation could advance patient-centered radiology while maintaining clinical accuracy, ultimately contributing to broader discussions about the role of AI in health care communication.

## Methods

### Radiology Report Collection and Anonymization

The electronic medical record was queried for radiology reports performed at our institution in September 2023. A total of 30 radiology reports were selected for analysis (6 brain magnetic resonance imaging, 6 brain computed tomography, 6 head and neck computed tomography angiography, 6 neck computed tomography, and 6 lumbar spine computed tomography) to reflect a broad range of imaging types and pathologies encountered in our neuroradiology section. Each radiology report was anonymized to remove all identifying information, including dates of comparison studies, if referenced.

### Patient-Centric Summary Creation

Each radiology report was processed by ChatGPT-4.0 (OpenAI), accessed via the web interface in October 2023, within a new chat dialogue to ensure consistent performance of the model across each report. No custom instructions or memory features were activated; thus, the prompt template for each report conversion followed a zero-shot prompt format, as follows:

*Please rewrite the following radiology report as a patient-friendly summary between 100 and 175 words. Start with a simple overall impression statement using natural language that is easy to understand. Focus on concisely summarizing the most relevant findings, conclusions, and recommended next steps. Minimize medical jargon, but clearly define any complex technical terms needed for accuracy. Provide enough details and context so that a layperson understands the key results and implications of the radiology report. Accurately reflect any potential abnormalities rather than omitting them. The summary should strike a balance between brevity and inclusion of significant information from the original report. The goal is an accessible and easy to understand summary that avoids oversimplification and remains clinically aligned with objective findings. Ensure the significance and severity of any abnormalities are accurately represented without downplaying or exaggerating. Only include next steps if you are highly confident they are appropriate recommendations for the patient.*

### Recruitment

Members of the study team (JMH and AS) visited our health system’s Patient and Family Advisory Council regularly scheduled meetings to outline the purpose and structure of our study and its focus on evaluating AI-generated summaries of radiology reports, and to ask for volunteers to participate. Four patients subsequently volunteered to complete survey questions about the reports and summaries. Four board-certified neuroradiologists were directly recruited by the study team (JMH) to complete survey questions on the quality and accuracy of the summaries from a single institution.

### Survey Design

The patient survey was a closed survey designed to separately evaluate patients’ understanding, satisfaction, and perceived utility of the radiology reports and summaries. The radiologist survey was a closed survey designed to assess the technical accuracy, clinical completeness, clarity, and potential errors that may have been introduced into the summary. Both surveys included 5-point Likert-scale questions and open-ended responses. Surveys were created and administered electronically to all study participants using Qualtrics software, with each participant being sent a unique link to prevent multiple submissions. In the survey, each report and summary was clearly labeled as “Original Radiology Report” and “ChatGPT Patient-Centric Summary,” respectively. The usability of the survey instruments was tested by the study team before deployment using Qualtrics. Complete survey instruments are provided in Multimedia Appendix 1.

### Word Count and Readability Analysis

Textual characteristics of the original radiology reports and patient-centric summaries were analyzed via word count and validated readability metrics. These included the Flesch-Kincaid Grade Level, Flesch Reading Ease score, Gunning-Fog Index, and SMOG (Simple Measure of Gobbledygook) Index, which provide estimates of the complexity and accessibility of the reports and summaries [22-24].

### Statistical Analysis

Differences in word count and readability scores between the reports and summaries were calculated with paired *t* tests using the Microsoft Excel Analysis ToolPak. For the survey questions, closed-ended responses were rated on Likert scales, with scores reported as percentages, and reflected as top 2 (or bottom 2) box scores as appropriate, aggregating responses from the Likert scale. Each patient and radiologist’s response for each report and summary was considered an individual data point.

Data from the open-ended questions were analyzed using a multifaceted qualitative approach. To capture a comprehensive understanding of patient and provider perspectives, the method of analysis was based on the structure of each question. During inductive analysis, open-ended responses were coded manually, sorting responses by found themes. To ensure that information was not lost or assumptions about implied importance, each response could be coded to multiple themes.

To determine how well patients understood the original radiology report and summary, their responses to the question “What do you believe to be the most important/main point that the radiologist was trying to convey?” were directly compared for congruence with answers supplied by the radiologist for each report/summary pair. Comparisons were then categorized as congruent or incongruent with the radiologist’s interpretation, which was considered the gold standard.

In addition, patient responses after reading the summary were compared to their answers after reading only the report. If the answers from the summary matched the answer from the report, responses were considered congruent (ie, the summary did not change the patient’s subjective understanding). If the answers did not match, responses were considered incongruent (ie, the summary did change the patient’s subjective understanding), with an additional positive or negative change modifier. A positive change modifier was assigned when responses included additional details or demonstrated closer alignment with the radiologist’s responses. Conversely, a negative change modifier was assigned when responses contained fewer details or diverged further from the radiologist’s responses.

### Ethical Considerations

This study evaluated the quality and understandability of ChatGPT-generated radiology report summaries through both quantitative and qualitative analyses via surveys. Ethics approval was granted by the Colorado Multiple institutional review board (23‐0731). All study participants were fully informed about this study, potential risks, and their right to withdraw at any time. All participants provided electronic informed consent. No compensation was provided to participants. All data were deidentified, and all analyses were conducted in accordance with data privacy guidelines.

## Results

### Readability

Patient-centric summaries demonstrated significant improvements in readability compared to original radiology reports across all measured metrics (Table 1). The summaries achieved a mean Flesch-Kincaid Grade Level of 7.2, representing a middle school reading level, compared to 12.5 for the original reports (*P*<.001). Similar improvements were observed in Flesch Reading Ease score, Gunning-Fog Index, and SMOG Index, consistently indicating enhanced accessibility of the summary content.

**Table 1.** Validated readability metric scores for the original radiology reports and ChatGPT-generated summaries.

Metric	Original report, mean (SD)	Summaries, mean (SD)	*P* value
Word count	241.0 (130.5)	189.0 (34.7)	.02
Flesch-Kincaid Grade Level	12.5 (1.63)	7.2 (1.14)	<.001
Flesch Reading Ease score	21.5 (11.39)	69.8 (6.2)	<.001
Gunning-Fog Index	16.4 (2.38)	10.3 (1.41)	<.001
SMOG^a^ Index	12.7 (1.55)	10.4 (1.05)	<.001

a SMOG: Simple Measure of Gobbledygook.

### Radiologist Survey

Four board-certified neuroradiologists completed surveys for each of the 30 radiology report/summary pairs, resulting in a total of 120 responses per question. The neuroradiologists had a mean of 11.6 (SD 9.33, range 3.5‐25) years of experience in neuroradiology after completing a fellowship.

Radiologist assessments of the summaries were generally favorable (Table 2). In 83% (100/120) of responses, radiologists indicated that the summaries represented the original reports extremely well/very well, and 90% (108/120) were satisfied with the balance of medical jargon to patient-centric language. Regarding accuracy, 5% (6/120) of responses indicated missing information, 12% (14/120) indicated overemphasis of findings, and 18% (22/120) indicated underemphasis. Notably, no hallucinations or fabricated findings were identified in any summary. Regarding the potential for patient misunderstanding, 82% (99/120) of radiologist responses indicated the summaries would likely not lead to incorrect conclusions, while 10% (12/120) indicated a perceived risk of misinterpretation.

**Table 2.** Radiologist assessment of the quality and accuracy of ChatGPT-generated summaries (N=120).

Measure and response	Value, n (%)
Representation of original findings	
Extremely well/very well	100 (83)
Neutral	14 (12)
Slightly well/not well at all	6 (5)
Satisfaction with the balance of medical jargon and patient-centric language	
Very satisfied/somewhat satisfied	108 (90)
Neutral	5 (4)
Somewhat dissatisfied/very dissatisfied	7 (6)
Error analysis	
Missing relevant information	6 (5)
Overemphasis of findings	14 (12)
Underemphasis of findings	22 (18)
Risk of leading patients to incorrect conclusions	
Definitely yes/probably yes	12 (10)
Neutral	9 (8)
Probably not/definitely not	99 (82)

In their free-text responses, neuroradiologists indicated several issues with the summaries. They noted an overemphasis on minor findings (eg, mild sinus disease, white matter changes, and renal cysts) and perceived some follow-up recommendations as unnecessary. Radiologists also reported disagreement with specific terminology choices in some cases (eg, using “mildly enlarged” to describe a “stable to incrementally” enlarged collection). They further noted that summaries underemphasized some critical findings, such as arterial occlusions, perfusion deficits, and nerve root compression, by not adequately conveying their clinical significance. In addition, radiologists expressed concerns that oversimplified vocabulary diminished the importance of certain findings, such as describing a vertebral artery as “a smaller artery in your neck” or referring to a normalized brain volume at the 6th percentile as “a bit below average.”

Qualitative analysis of radiologist feedback revealed several themes regarding desired improvements to the summaries. Fifty-four percent (66/122) of free-text responses indicated no improvements were needed. In 38% (46/122), radiologists expressed discomfort with the perceived oversimplification of language (eg, “lump” instead of “nodule,” “thinning” instead of “osteopenia,” “squishing” instead of “height loss”), and generally found the summaries could have been more concise. In 4% (5/122) of responses, radiologists indicated a preference for more detail, including more clearly defined terminology and more explicit localization of relevant findings (eg, the level of spinal stenoses).

### Patient Survey

Four patient volunteers completed surveys for each of the 30 report-summary pairs, resulting in a total of 120 responses across all 30 reports and summaries for each question. A total of 118 responses were valid, with the remaining 2 responses left blank. Patients’ reported age ranged between 55 and 84 years: 1 (55-64 years), 2 (65-74 years), and 1 (75-84 years). Patients’ reported educational attainment included high school graduate (n=1), college degree (n=2), and master’s degree (n=1).

Patient survey responses indicated substantially improved subjective comprehension and satisfaction with summaries compared to the original reports (Table 3). There were large, highly significant improvements in patient ratings when reading the summary versus the original report (*P*<.001). While only 26% (31/118) of responses indicated confidence in understanding the original reports, this increased to 98% (116/118) of responses for summaries. Satisfaction with medical terminology showed similar improvement, rising from 8% (9/118) for original reports to 91% (107/118) for summaries. Time required for comprehension was a significant concern with original reports, with only 23% (27/118) satisfied with the time needed for understanding, compared to 97% (115/118) satisfaction with summaries.

**Table 3.** Patient confidence and satisfaction with the original radiology reports and ChatGPT-generated summaries (N=118).

Measure and response	Original report, n (%)	Summaries, n (%)	*P* value
Confidence in understanding			<.001
Very confident/somewhat confident	31 (26)	116 (98)	
Neutral	14 (12)	1 (1)	
Very unconfident/somewhat unconfident	73 (62)	1 (1)	
Satisfaction with medical jargon/terminology			<.001
Very satisfied/somewhat satisfied	9 (8)	107 (91)	
Neutral	18 (15)	9 (8)	
Very dissatisfied/somewhat dissatisfied	91 (77)	2 (2)	
Satisfaction with level of detail			<.001
Very satisfied/somewhat satisfied	51 (43)	116 (98)	
Neutral	43 (36)	1 (1)	
Very dissatisfied/somewhat dissatisfied	24 (20)	1 (1)	
Satisfaction with time to comprehension			<.001
Very satisfied/somewhat satisfied	27 (23)	115 (97)	
Neutral	31 (26)	2 (2)	
Very dissatisfied/somewhat dissatisfied	60 (51)	1 (1)	

The summaries demonstrated high utility, with 92% (108/118) of responses indicating they clarified questions about the original report and improved patients’ understanding of clinical implications. When asked about format preferences, 67% (79/118) of responses indicated a preference for having access to both the summary and original report, while 26% (31/118) of responses preferred to have the summary alone. Only 1% (1/118) preferred the original report exclusively, with the remaining 6% (7/120) indicating a preference for alternative formats.

Qualitative analysis of patient feedback revealed several themes regarding desired improvements to the summaries. Of the free-text responses, 50% (53/107) indicated no improvements were needed. The remaining responses suggested enhancing the professional tone (13%, 14/107), providing more specific follow-up recommendations (14%, 15/107), and including additional detail about medical terminology and physiological processes relevant to the findings (23%, 25/107).

### Effect of the Summary on Patient Understanding

Patient understanding was assessed by comparing each patient’s identification of the main point of the report/summary against the radiologist’s response regarding the main point of the report. When reading the original radiologist report, 60% (71/119) of patient responses aligned with radiologist interpretations, while 40% (48/119) were incongruent. Patient understanding improved with the summary, with 74% (88/119) of responses aligning with radiologist interpretations, while 27% (32/119) remained incongruent.

When comparing patient responses regarding the main point the radiologist was trying to convey after reading only the report with their response after reading the summary, answers did not change in 52% (62/119) (ie, responses were congruent). In 29% (34/119), the responses after reading the summary were different (ie, the summary changed a patient’s understanding). Specifically, in 23% (27/119), responses after reading the summary contained additional relevant details or demonstrated closer alignment with radiologists’ responses, suggesting an increase in understanding as a result of the summary. In 6% (7/119), responses after reading the summary contained fewer details or diverged further from radiologists’ responses, suggesting decreased understanding as a result of the summary.

## Discussion

### Principal Findings

This study demonstrates that LLMs can successfully generate neuroradiology reports that may be more understandable to patients while maintaining clinical accuracy. The AI-generated summaries significantly improved readability metrics across all measured scales, reducing the required reading level from college (grade 12.5) to middle school (grade 7.2), while substantially increasing reading ease scores [25,26]. This improvement in accessibility was achieved while maintaining high accuracy, with radiologists confirming that 83% (100/120) of summaries represented the original findings “extremely” or “very well.” Similar studies have shown that such summaries maintain high accuracy [27,28].

A unique strength of this study is the inclusion of patients in the evaluation of reports and AI-generated summaries, as the vast majority of research focuses on evaluation by radiologists and nonradiologist physicians [2,29-31]. Patient response data revealed a striking contrast between comprehension of traditional reports and AI-generated summaries, with only 26% (31/118) of patients reporting confidence in understanding the original reports and 98% (116/118) reporting confidence in understanding the summaries. In addition, the high satisfaction rates with the summaries’ level of detail (98%, 116/118) and medical terminology (91%, 107/118) indicate that the AI successfully struck a balance between accessibility and informativeness.

The dramatic improvement in patient confidence was accompanied by objective improvements in understanding, with congruence between patient and radiologist interpretations increasing from 60% (71/119) on the original reports to 74% (88/119) after reading the summaries. In addition, 23% (27/119) of responses after reading the summary contained additional relevant details or demonstrated closer alignment with radiologist responses, consistent with an increased understanding as a result of the summary. This suggests that AI-generated summaries not only increase patient confidence but also lead to a more accurate understanding of radiological findings. This aligns with prior evidence that patient-friendly summaries can enhance patient understanding; for example, nonphysician readers in one study had comprehension scores of 4.69/6 for AI-generated summaries versus 2.71/5 for original reports [27].

Particularly noteworthy was the strong patient preference for having access to both the original report and the summary (67%, 79/118 of responses), with 98% (116/118) of responses indicating they would use the summary if available. This suggests that patients value the combination of detailed technical information with accessible explanations, rather than seeing the summary as a replacement for the full report. These findings align with the shifting preference among the general public toward greater transparency in health care [3,4,7,8].

Importantly, no frank hallucinations were observed in the summaries, which is crucial for clinical implementation. However, a small percentage (10%, 12/120) of radiologist responses indicated that summaries may be misleading. Radiologists noted instances of omission and both over- and underemphasis of findings (12%, 14/120 and 18%, 22/120 of responses, respectively), particularly regarding incidental findings and critical abnormalities. The concern about misleading summaries was corroborated by the 6% (7/119) of patient responses that contained fewer details or diverged further from radiologist responses after reading the summary. Misleading information has the potential to lead to a variety of negative consequences for the patient, further emphasizing the need for comprehensive standards surrounding LLM-generated resources [20].

In addition, in some instances, language was deemed too casual by radiologists (eg, “squished” instead of “compressed”), highlighting the need for more sophisticated prompt engineering to maintain professional medical communication standards while improving accessibility. An area of improvement identified by the patient reviewers was highlighted by the 14% (15/107) of patient responses indicating a desire for more explicit guidance on next steps and implications of findings.

### Limitations

This study has several limitations. Principally, its focus on neuroradiology reports from a single institution potentially limits the generalizability of our findings to other radiologic subspecialties and other institutional reporting patterns. In addition, the study included a relatively small number of patient and radiologist reviewers. We hope that this work encourages other investigators to replicate this study with larger cohorts of reviewers to assess its applicability in subspecialties outside of neuroradiology and in larger and more diverse patient populations. Patient volunteers were relatively older and had a relatively higher average education level than the general population and were members of our health institution’s Patient Family Advisory Council. This likely indicates a higher level of health literacy and reading ability compared to the average patient. Overall, we anticipate that if anything, this biased the results toward greater understanding of the original radiology report, rather than less. Finally, volunteers were not reading their own reports, which removes the emotional salience and may alter the experience.

### Considerations for Clinical Deployment

Clinical implementation of AI-generated summaries of radiology reports raises a number of ethical and practical considerations that must be addressed. In reference to ChatGPT, OpenAI policy explicitly requires that AI-generated medical content undergo review by qualified professionals due to the risk of AI errors, which would necessitate radiologist or ordering provider oversight of all summaries before patients can access them [32]. The choice of model is incredibly important, as different models perform differently on such summarization tasks [28]. Clinical deployment requires HIPAA (Health Insurance Portability and Accountability Act)-compliant platforms (many are already available) and integration into existing electronic health record workflows without excessive burden on providers. Given the extraordinary burden already placed on radiologists to complete standard dictations amid increasing imaging volumes, adding review of an AI-generated summary to the workflow after final signature may not be feasible. It is important to note, however, that patients are already using consumer AI tools/chatbots to interpret their medical records without professional oversight, potentially receiving inaccurate or anxiety-provoking information [33]. Institution-supervised AI summaries could provide a safer alternative to this unregulated landscape while ensuring some degree of professional oversight and quality assurance. Future implementations must balance the demonstrated benefits of improved patient comprehension with robust safeguards.

### Future Research

AI-generated, patient-friendly summaries appear to represent a promising tool for improving patient understanding of radiology reports. The summaries had high accuracy rates and dramatically improved readability and patient comprehension while maintaining clinical precision. The perceived benefit to patients in conjunction with the objective increase in comprehension suggests potential benefit in terms of patient experience and outcomes, though further investigation is needed using patients’ own scans and reports (which we are pursuing) [1,3,8,10-12]. Future work should also investigate how prompt optimization and custom model development may improve the quality of outputs. Integration of these summaries into existing radiology workflows and patient portals should be studied, with particular attention to the ultimate impact on radiologist workload, patient experience, and outcomes. Conscientious integration of this technology is required, given the extraction of sensitive patient data and associated concerns [34].

Future research should explore options to continue refining the clinical accuracy of these reports. Though LLMs lack the ability to independently reason, there are several ways to optimize their output, including optimization of prompt engineering, incorporating structured templates for critical findings, and using standardized language for common pathologies [2,30,31]. In addition, providing the LLM with more clinical context (eg, recent note from an ordering provider, problem list, patient class [IP/OP/ED]) may improve the quality of the summary and recommendations provided [31,35].

### Conclusions

This study demonstrates that ChatGPT-generated summaries can help bridge the communication gap between complex radiology reports and patient understanding while maintaining accuracy. While promising, our findings highlight the need for continued refinement to address instances of overemphasis or underemphasis, which can mislead patients in certain circumstances. As such, it is critically important to establish robust quality assurance frameworks before clinical implementation.

## Supplementary material

10.2196/76097 Multimedia Appendix 1 Complete patient and radiology survey instruments.

## Abbreviations

AI: artificial intelligence
HIPAA: Health Insurance Portability and Accountability Act
LLM: large language model
SMOG: Simple Measure of Gobbledygook
