# Supplementary material for: Evaluating the Quality and Understandability of Radiology Report Summaries Generated by ChatGPT: Survey Study
Source: JMIR Form Res. 2025 Aug 27;9:e76097. doi: 10.2196/76097 (PMC12385610; doi:10.2196/76097)
Supplement: Multimedia Appendix 1 [file formative-v9-e76097-s001.docx]

**Patient Survey Instrument**
Please read the original radiology report and then answer the following questions:

1. How confident are you in your understanding of the original radiology report?
2. Very Unconfident, Somewhat Unconfident, Neutral, Somewhat Confident, Very Confident
3. How satisfied are you with the amount of time it took to feel you understood the original radiology report?
4. Very Dissatisfied, Somewhat Dissatisfied, Neutral, Somewhat Satisfied, Very Satisfied,
5. How satisfied are you with the level of detail provided in the original radiology report?
6. Very Dissatisfied, Somewhat Dissatisfied, Neutral, Somewhat Satisfied, Very Satisfied
7. How satisfied are you with the amount of medical jargon/terminology present in the original radiology report?
8. Very Dissatisfied, Somewhat Dissatisfied, Neutral, Somewhat Satisfied, Very Satisfied
9. What do you believe to be the most important / main point that the radiologist was trying to convey in the original radiology report?
10. ______________________________________________

Please read the patient centric summary report and then answer the following questions:

1. How confident are you in your understanding of the patient-centric summary of the report?
2. Very Unconfident, Somewhat Unconfident, Neutral, Somewhat Confident, Very Confident
3. How satisfied are you with the amount of time it took to feel you understood the patient-centric summary of the report?
4. Very Dissatisfied, Somewhat Dissatisfied, Neutral, Somewhat Satisfied, Very Satisfied
5. How satisfied are you with the level of detail provided in the patient-centric summary of the report?
6. Very Dissatisfied, Somewhat Dissatisfied, Neutral, Somewhat Satisfied, Very Satisfied
7. How satisfied are you with the amount of medical jargon/terminology present in the patient-centric summary of the report?
8. Very Dissatisfied, Somewhat Dissatisfied, Neutral, Somewhat Satisfied, Very Satisfied
9. Please write down what you believe to be the most important / main point that the radiologist was trying to convey based on the patient-centric summary of the report?
10. ______________________________________________
11. What changes would you make to the patient-centric summary of the report to improve it?
12. ______________________________________________

Please answer the following questions based on your impressions of both the original report and the summary?

1. If this patient-centric summary was available to read alongside the original report in your health record, how likely would you be to utilize it?
2. Very Unlikely, Somewhat Unlikely, Neutral, Somewhat Likely, Very Likely
3. What would you like to have available to you for your own radiology imaging studies?
4. Only the Original Report, Only the Patient-Centric Summary, Both Versions, Something Else
5. Did the patient-centric summary clarify questions you had regarding the original report?
6. Definitely Not, Probably Not, Neutral, Probably Yes, Definitely Yes
7. Did the summary help you better understand the implications to your health?
8. Definitely not, probably not, Neutral, Probably Yes, Definitely Yes.
9. Please provide any additional insight / comments you may have about the original report and patient-centric summary that might have been missed on this survey.
10. _____________________________________

**Radiologist Survey Instrument**

Please read the original radiology report and summary and then answer the following questions:

1. How well does the patient-centric summary represent the original report?
2. Not at all, Slightly, Moderately, Very, Extremely
3. How satisfied are you with the balance between medical jargon and patient centric language in the patient-centric summary?
4. Very dissatisfied, Somewhat Dissatisfied, Neutral, Somewhat Satisfied, Very Satisfied,
5. How satisfied are you with the clarity of the patient-centric summary?
6. Very dissatisfied, Somewhat Dissatisfied, Neutral, Somewhat Satisfied, Very Satisfied
7. How well does the patient-centric summary maintain the medical accuracy of the original report?
8. Not at all, Slightly, Moderately, Very, Extremely
9. Were any relevant medical information/important findings missed in the summary? If Yes, please specify.
10. _________________________________________________
11. Were any relevant medical information/important findings underemphasized in the summary? If yes, please specify.
12. _________________________________________________
13. Were any relevant medical information/important findings overemphasized in the summary? If yes, please specify.
14. _________________________________________________
15. Does the summary lead patients to incorrect conclusions about the report and their health?
16. Definitely Not, Probably Not, Might or Might Not, Probably Yes, Definitely Yes
17. If Yes, Please provide examples of this ____________________________
18. Please write down in your own words what you believe to be the most important / main point that the radiologist was trying to convey to the patient in the original radiology report?
19. _____________________________________________
20. What changes, if any, would improve the patient-centric summary of the report?
21. ______________________________________________
